# Changes in uric acid metabolism and associated plasma proteomics during sex hormone therapy

**DOI:** 10.1016/j.jcte.2026.100434

**Published:** 2026-02-27

**Authors:** Sarah A. van Eeghen, Danii L.S. Suijk, Laura L. Pyle, Phoom Narongkiatikhun, Ye Ji Choi, Natalie J. Nokoff, Petter Bjornstad, Martin den Heijer, Daniël H. van Raalte

**Affiliations:** a Center of Expertise on Gender Dysphoria, Department of Internal Medicine, Amsterdam University Medical Center, Location VU Medical Center, Amsterdam, the Netherlands; b Amsterdam Gastroenterology Endocrinology Metabolism, Amsterdam University Medical Center, Amsterdam, the Netherlands; c Department of Endocrinology and Metabolism, Amsterdam University Medical Center, Location VU Medical Center, Amsterdam, the Netherlands; d Amsterdam Cardiovascular Sciences, VU University, Amsterdam, the Netherlands; e Department of Medicine, Division of Endocrinology, Metabolism and Nutrition, University of Washington School of Medicine, Seattle, WA, USA; f Department of Pediatrics, Section of Endocrinology, University of Colorado School of Medicine, Aurora, CO, USA; g Division of Nephrology, Department of Internal Medicine, Faculty of Medicine, Chiang Mai University, Chiang Mai, Thailand; h Diabetes Center, Amsterdam University Medical Center, Location VU Medical Center, Amsterdam, the Netherlands

**Keywords:** Sex hormones, Uric acid, Proteomics, CKM syndrome

## Abstract

**Introduction:**

Men generally have higher plasma uric acid (PUA) concentrations and are at greater risk for hyperuricemia-related diseases. Sex hormones may underlie this difference, though mechanisms remain unclear. Sex hormone therapy offers a unique opportunity to study these effects.

**Methods:**

Participants were included from two prospective observational cohorts: ENIGI (n = 544, 12 months of feminizing [n = 260] or masculinizing [n = 284] hormone therapy) and KNIGHT (n = 28, 3 months of feminizing [n = 15] or masculinizing [n = 13] hormone therapy). Outcomes included changes in PUA (ENIGI), uric acid clearance (UAC; KNIGHT), fractional excretion of uric acid (FE-UA; KNIGHT), fat distribution (ENIGI), and PUA-associated plasma proteomics (KNIGHT).

**Results:**

PUA decreased during feminizing hormone therapy (−86  μmol/L [95% CI, −95; −77]) and increased during masculinizing hormone therapy (+61  μmol/L [95% CI, 53; 70]). During feminizing therapy, UAC increased (+1.1  mL/min [95% CI, 0.1; 2.1]) without changing FE-UA. During masculinizing therapy UAC and FE-UA decreased (UAC: −1.0  mL/min [95% CI, −1.7; −0.3]; FE-UA: −0.8% [95% CI, −1.3; −0.4]). Android-to-gynoid and visceral-to-subcutaneous fat ratios decreased with feminizing and increased with masculinizing therapy (p < 0.05) and correlated positively with PUA changes (p < 0.001). Proteomics identified 126 PUA-associated proteins differentially expressed during sex hormone therapy. While proteins directly involved in uric acid production were unchanged, proteins related to fat distribution (adiponectin, leptin, SHBG) associated with PUA changes.

**Discussion:**

PUA significantly changes during sex hormone therapy, most likely through changes in UAC and fat distribution. These findings emphasize the role of sex hormones in uric acid metabolism and provide insight into sex differences in hyperuricemia-related conditions.

**Trial registration knight:**

Dutch Trial Register (ID: NL9517); ClinicalTrials.gov (ID: NCT04482920).

## Highlights

• Plasma uric acid (PUA) decreases with feminizing; increases with masculinizing therapy.
• Uric acid clearance increases with feminizing; decreases with masculinizing therapy.
• These changes are associated with changes in uric acid clearance and fat distribution.
• Plasma proteomics show fat distribution-related proteins correlate with PUA changes.

## Introduction

Elevated plasma uric acid (PUA) is consistently associated with a higher risk of cardiovascular, metabolic, and kidney diseases [1], [2], [3], which are increasingly recognized as interconnected conditions within the cardiovascular-kidney-metabolic (CKM) syndrome [4]. Approximately 30% of the global population is affected by CKM-related multimorbidity, underscoring the need for shared biomarkers and therapeutic targets [4]. Given its broad range of associations, uric acid has emerged as a promising candidate [2], [3], [5].

PUA concentrations differ markedly by sex, with lower concentrations typically observed in women–a difference that becomes apparent at puberty and attenuates after menopause [6], [7]. This suggests that sex hormones, particularly estradiol and testosterone, play key roles in regulating uric acid metabolism [6], [7]. Importantly, sex hormones may also influence the risk of CKM syndrome, with premenopausal women showing relative protection against CKM-related conditions compared with men [8], [9], [10], [11], [12], [13]. Together, these observations support the hypothesis that sex hormone-dependent regulation of uric acid metabolism may contribute to sex disparities in CKM syndrome prevalence and severity.

Despite these associations, the mechanisms through which sex hormones influence uric acid metabolism are incompletely understood. It remains unclear whether these effects occur via direct modulation of uric acid production or kidney excretion, or indirectly through changes in body composition or fat distribution [14], [15]. This gap in mechanistic understanding limits progress toward sex- and sex hormone-informed precision medicine strategies aimed at reducing PUA in the context of CKM-related conditions.

Research involving transgender individuals undergoing sex hormone therapy provides a unique opportunity to examine the physiological effects of sex hormones on uric acid metabolism. Moreover, with the rising number of transgender individuals receiving sex hormone therapy and emerging evidence of their increased vulnerability to CKM-related diseases, this research is of particular importance for their health and clinical care [16]. However, existing studies in this population are limited by small sample sizes and a lack of mechanistic insight [17], [18], [19].

To address these gaps, we assessed changes in PUA concentrations, uric acid clearance (UAC), fractional excretion of uric acid (FE-UA), body composition, fat distribution, and uric acid-related proteomics in transgender adults before and during 3–12 months of hormone therapy.

## Materials and methods

### Participants and study design

This study included participants from two complementary prospective observational cohorts to capture both clinical and mechanistic dimensions of uric acid metabolism: the large scale European Network for the Investigation of Gender Incongruence (ENIGI) and the mechanistic Kidney fuNction In people receiving Gender Affirming Hormone Therapy (KNIGHT) study. Both received ethical approval, with written informed consent, in accordance with the Declaration of Helsinki and Good Clinical Practice. The KNIGHT study is registered at the Dutch Trial Register (NL9517) and clinicaltrials.gov (NCT04482920).

#### ENIGI

ENIGI is a multicenter study examining the effects of sex hormone therapy in transgender individuals since 2012 [20]. For this analysis, participants from the ENIGI study were selected based on specific criteria. Firstly, only individuals from the Amsterdam University Medical Center (Amsterdam UMC) were included. Sample size calculations, based on previous data on PUA changes during sex hormone therapy, indicated that 62 feminizing and 49 masculinizing participants would provide 90% power at a 5% significance level [19]. Given the extensive cohort at Amsterdam UMC, including only individuals from this center was sufficient. Secondly, inclusion required baseline and 12-month follow-up visits. Thirdly, since PUA is also shown to be associated with body composition and fat distribution [21], [22], which significantly change during sex hormone therapy [23], participants with body composition measurements (bioelectrical impedance analysis [BIA] or whole body dual-energy x-ray absorptiometry [DXA]) at both visits were included. Participants using antiandrogens other than cyproterone acetate (CPA) were excluded. The final cohort included 260 individuals on feminizing and 284 individuals on masculinizing hormone therapy (June 2012–August 2019; Supplemental Fig. 1). Due to incompatibility between DXA and BIA, body composition analyses included only participants with DXA at both visits.

#### KNIGHT

KNIGHT is a prospective observational study conducted at Amsterdam UMC and the University of Colorado Anschutz Medical Campus (CU-AMC) from April 2021 to June 2023. Of 44 participants completing both study visits (including plasma proteomics); 29 participants from Amsterdam UMC who completed 24-hour urine collections were included in this analysis: 16 receiving feminizing and 13 receiving masculinizing hormone therapy (Supplemental Fig. 2). These participants were recruited through the Amsterdam UMC Center of Expertise on Gender Dysphoria outpatient clinic.

Study visits occurred just before and 3 months after sex hormone therapy initiation. The sample size was based on the KNIGHT study’s primary objective, reported elsewhere [24]. Accordingly, the KNIGHT cohort was not specifically powered to detect changes in plasma uric acid (PUA), uric acid clearance (UAC), or fractional excretion of uric acid (FE-UA), and is therefore interpreted primarily as a mechanistic cohort.

Eligible participants were aged 17–40 years, diagnosed with gender dysphoria (DSM-V), and initiating sex hormone therapy within one month. Exclusion criteria included cognitive, psychiatric, or physical impairments; current or prior use of sex hormones or antiandrogens; gonadectomy; pregnancy; participation in other studies; antihypertensive medication use; kidney disease (eGFR < 60 ml/min per 1.73 m^2^ or urine albumin-to-creatinine ratio > 2.5 mg/mmol); diabetes mellitus; uncontrolled hypertension; cardiovascular disease; or iodine allergy.

### Treatment protocols

#### ENIGI

Feminizing hormone therapy included oral estradiol valerate (4 mg/day), transdermal patches (50–100 µg/24 h), or gel (1.5 mg/day), with CPA (10–100 mg/day) as antiandrogen. Masculinizing hormone therapy included transdermal testosterone gel (37.5–50 mg/day), intramuscular testosterone undecanoate (1000 mg/12 weeks), or intramuscular testosterone blend (comprising a blend of 30 mg testosterone propionate, 60 mg phenylpropionate, 60 mg isocaproate, and 100 mg decanoate; 250 mg/2–3 weeks).

#### KNIGHT

Feminizing hormone therapy included oral estradiol (4 mg/day) or transdermal estradiol via a patch (100 µg/24 h), combined with intramuscular triptorelin (3.75 mg every 4 weeks). Masculinizing hormone therapy included testosterone gel (40 mg/day) or intramuscular testosterone blend (250 mg/3 weeks).

### Data collection

#### ENIGI

Fasting venous blood was collected at baseline and 12 months for sex hormones, creatinine, cystatin C, and PUA. Whole body DXA (Hologic Discovery 13.1, which was updated to version 3.3 in 2012 and to version 4.5.3 in 2015) was used to assess lean body mass, and total, android, gynoid, visceral and subcutaneous fat mass as described in previous ENIGI manuscripts [23], [25]. Android-to-gynoid fat ratio was calculated as android fat mass (kg) divided by gynoid fat mass (kg), and visceral-to-subcutaneous fat ratio was calculated as visceral fat (kg) divided by subcutaneous fat (kg).

#### KNIGHT

At least three days prior to both study visits, participants adhered to “normal” sodium (9–12 g/d) and protein (1.5–2.0 g/kg/d) diets, and were asked to refrain from vigorous physical activity and alcohol ingestion for at least 24 h, and from consuming caffeine for at least 12 h. Fasting venous blood samples and 24-hour urine samples were collected at baseline and 3 months. Venous blood samples for sex hormones, creatinine, cystatin C, PUA, and plasma proteomics, and 24-hour urine samples for creatinine, and uric acid.

Detailed instructions were provided verbally and in writing regarding the collection of the 24-hour urine samples, including refraining from strenuous exercise, using dedicated containers, and storing samples in a refrigerator until delivery to the clinic. The 24-hour urine collection began the day before the study visit after the first morning urine and concluded on the day of the visit including the first morning urine.

UAC was calculated using the equation: UAC = (1000 × uric acid in 24-hour urine sample (umol/L) /PUA (umol/L)) × (24-hour urine volume (mL) / 1440). FE-UA was calculated using the equation: FE-UA = (uric acid in 24-hour urine sample (umol/L) * serum creatinine (umol/L)) / (PUA * creatinine in 24-hour urine sample (umol/L)) * 100.

### Laboratory measurements

Initially, estradiol was measured by competitive immunoassay (Delfia; PerkinElmer, Turku, Finland) with an interassay coefficient of variation (CV) range of 10%–13% and a limit of quantitation (LOQ) of 5.45 pg/ml. Subsequently liquid chromatography–tandem mass spectrometry (LC-MS/MS) was employed in July 2014, with an interassay CV of 7% and an LOQ of 5.45 pg/ml. A conversion formula (LC-MS/MS = 1.60 × Delfia − 29) was applied to align Delfia values with LC-MS/MS results. Until January 2013, testosterone was measured using radioimmunoassay (RIA; Coat-A-Count; Siemens, Los Angeles, CA) with an interassay CV of 7%–20% and an LOQ of 29 ng/dl. After this period, a competitive immunoassay (Architect; Abbott, Abbott Park, IL) was utilized, with an interassay CV range of 6%–16% and an LOQ of 2.9 ng/dl. Conversion formulas were applied for alignment: for testosterone < 231 ng/dl, Architect = 1.1 × RIA + 0.2, and for testosterone > 231 ng/dl, Architect = 1.34 × RIA − 1.65. From October 2018, LC-MS/MS was used for testosterone measurements, with an interassay CV of 4%–9% and an LOQ of 0.1 nmol/L. There was no need for a conversion formula.

PUA was measured via colorimetric assay (Roche/Hitachi cobas c311). A particle-enhanced immunoturbidimetric assay on a cobas analyzer (Roche cobas 8000 module c502, Roche Diagnostics, Mannheim, Germany) was used to measure serum cystatin C. Serum creatinine concentrations were measured using an enzymatic immunoassay performed on a cobas c system (Roche/Hitachi by Roche Diagnostics Mannheim, Germany).

### Plasma proteomics

Plasma protein concentrations at baseline and at 3-month follow-up were measured using the SOMAscan 7 K Proteomic platform (SomaLogic, Inc.) at Washington University in St. Louis, Missouri, USA [24], targeting 6,596 proteins with 7,604 aptamers [26]. Each sample included internal controls and was normalized to account for both intra- and inter-plate variation.

### Statistical analysis

#### Baseline characteristics and clinical outcomes

Analyses were performed in STATA® (version 17.0). Data were reported as absolute numbers (n), percentages (%), mean ± standard deviation (SD) for normally distributed data, or median with interquartile range (IQR) for non-normal distributions evaluated via visual inspection of histograms and comparing the mean (±SD) with the median (IQR).

Changes from baseline to follow up were assessed separately for feminizing and masculinizing hormone therapy, using Wilcoxon signed-rank tests (for sex hormone concentrations) and linear mixed models (for body mass index [BMI], blood pressure, creatinine, cystatin C, PUA, UAC, FE-UA, body composition and fat distribution). Normality was evaluated via visual inspection of histograms of the residuals. Sensitivity analyses for evaluating change in PUA excluded participants reporting current smoking or using other drugs affecting PUA. Linear mixed models adjusted for BMI, blood pressure, body composition, android-to-gynoid fat ratio, alcohol consumption, and kidney function changes, each in separate models.

Spearman’s rank correlations assessed associations between delta sex hormones and delta clinical outcomes (PUA, FE-UA and UAC). Additionally, correlations between delta android-to-gynoid and visceral-to-subcutaneous fat ratios, and delta PUA were analyzed. Delta represents the absolute change from baseline to follow-up. These analyses included participants undergoing masculinizing and feminizing hormone therapy as a combined group.

Administration route comparisons in ENIGI were restricted to participants using a consistent route over 12 months (n = 441).

#### Plasma proteomics

Proteomic data were analyzed using R (version 4.4.0, R Core Team, Vienna). Before analysis, protein measurements were log-transformed and standardized by dividing each value by the sample-specific standard deviation for that protein. Differential expression was assessed using linear models with moderated *t*-statistics [27], separately for masculinizing and feminizing hormone therapy. These differentially expressed proteins are presented in detail elsewhere [24].

Based on literature [28], [29], [30], we identified seven proteins directly involved in uric acid production; adenosine deaminase (ADA), purine nucleoside phosphorylase (PNP), guanine deaminase (GUAD), xanthine dehydrogenase/oxidase (XDH), hypoxanthine–guanine phosphoribosyltransferase (HPRT), ribose-phosphate pyrophosphokinase 1 (PRPS1), and adenine phosphoribosyltransferase (APT), and examined whether their expression changed during feminizing or masculinizing hormone therapy. Key urate transporters involved in kidney and intestinal excretion of uric acid could not be assessed using plasma proteomics, due to their membrane-bound nature and lack of presence in the circulation.

Further analyses were conducted to identify proteins specifically associated with delta PUA by assessing correlations between protein changes and delta PUA using Spearman’s rank correlation. We identified proteins that correlated with delta PUA across the full cohort and then refined our focus to proteins that changed during feminizing or masculinizing hormone therapy. A Venn diagram summarizing these identified proteins is provided in Supplemental Fig. 3.

Only participants with complete data for all relevant variables were included in the analysis. No imputation was performed for missing data.

P-values were adjusted to maintain a false discovery rate of 5%.

## Results

**Fig. 1:**
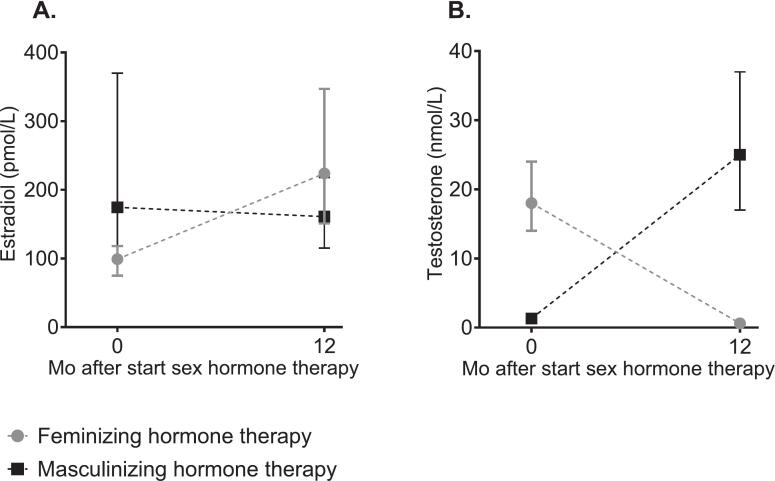
Serum estradiol and testosterone changed during 1 year of sex hormone therapy in the participants of the ENIGI study. Median with IQR of serum estradiol (A) and serum testosterone (B) before and at 12 months of feminizing and masculinizing hormone therapy. Abbreviations: IQR, interquartile range. Alt text: Graphs A and B show median (IQR) serum estradiol (pmol/L) and testosterone (nmol/L) concentrations, respectively, at baseline and 12 months after starting feminizing or masculinizing hormone therapy.

Baseline characteristics were comparable between cohorts (Table 1). Administration routes are listed in Supplemental Table 1. Sex hormone concentrations before and during sex hormone therapy are shown in Fig. 1 for ENIGI participants and were similar across the two cohorts (Supplemental Table 2).

**Table 1:** Baseline characteristics of the included participants.

Variable	ENIGI cohort		KNIGHT cohort	
	Individuals scheduled to start feminizing hormone therapy (n = 260)	Individuals scheduled to start masculinizing hormone therapy (n = 284)	Individuals scheduled to start feminizing hormone therapy (n = 15)	Individuals scheduled to start masculinizing hormone therapy (n = 13)
Age, year	29 (23–43)	22 (20–28)	26 (21–31)	21 (20–24))
Current smoker, n (%)	62 (24)	81 (29)	3 (20)	5 (38)
Alcohol consumption, units/week	0.05 (0–2)	0 (0–2)	1 (0–1)	1 (0–1)
BMI, kg/m^2^	24.0 (±4.8)	26.0 (±6.0)	27.2 (20.5–35.2)	23.9 (19.6–30.0)
Testosterone, nmol/L	18.0 (14.0–24.0)	1.3 (0.9–1.6)	15.0 (8.4–20)	1.0 (0.8–1.1)
Estradiol, pmol/L	99 (75–118)	175 (76–370)	74 (60–94)	137 (93–302)
Systolic BP, mmHg	129 (±13)	124 (±12)	117 (±9)	102 (±8)
Diastolic BP, mmHg	80 (±9)	76 (±9)	73 (±8)	67 (±7)
Creatinine, μmol/L	78.5 (±9.9)	66.7 (±9.3)	76.3 (±11.3)	61.7 (±8.1)
Cystatin C, mg/L	0.94 (±0.16)	0.90 (±0.17)	0.92 (±0.11)	0.79 (±0.08)
Plasma uric acid, μmol/L	365 (±77)	295 (±72)	373 (±60)	271 (±53)

Categorical variables are presented as n (%). Otherwise indicated, data are presented according to their distribution, median (interquartile range), and mean (±SD). Abbreviations: BMI, body mass index; BP, blood pressure; ENIGI, European Network for the Investigation of Gender Incongruence; KNIGHT, Kidney fuNction In people receiving Gender-affirming Hormone Therapy.

### Blood pressure, kidney function, body composition and fat distribution

Changes in BMI, blood pressure, cystatin C, and creatinine among ENIGI participants are summarized in Table 2 and described previously [31]. During feminizing hormone therapy, lean body mass decreased by 2.4% (95% CI, 1.5 to 3.3) and total fat mass increased by 26.2% (95% CI, 21.9 to 31.4, n = 100), accompanied by decreases in the android-to-gynoid fat ratio (−0.06; 95% CI, −0.07 to −0.05), and the visceral-to-subcutaneous fat ratio (−0.07; 95% CI, −0.08 to −0.05). Conversely, during masculinizing hormone therapy, lean body mass increased by 10.1% (95% CI, 8.9 to 11.2) and total fat mass decreased by 9.7% (95% CI, −12.3 to 7.0, n = 105), while the android-to-gynoid and visceral-to-subcutaneous fat ratios increased by 0.06 (95% CI 0.05 to 0.07, n = 104), and 0.008 (95% CI, 0.001 to 0.014), respectively.

**Table 2:** Measurements before and during sex hormone therapy.

	During feminizing hormone therapy		During masculinizing hormone therapy	
ENIGI	*Baseline*	*12 mo of sex hormone therapy*	*Baseline*	*12 mo of sex hormone therapy*
BMI, kg/m^2^	24.0 (±4.8)	24.6 (±4.2)^a^	26.0 (±6.0)	26.2 (±5.4)^b^
Systolic BP, mmHg	129 (±13)	127 (±12)^b^	124 (±12)	124 (±12)
Diastolic BP, mmHg	80 (±9)	79 (±9)^c^	76 (±9)	77 (±8)
Creatinine, μmol/L	78.5 (±9.9)	72.5 (±9.7)^a^	66.7 (±9.3)	78.3 (±10.5)^a^
Cystatin C, mg/L	0.94 (±0.16)	0.87 (±0.15)^a^	0.90 (±0.17)	0.95 (±0.16)^a^
PUA, μmol/L	365 (±77)	279 (±66)^a^	295 (±72)	357 (±76)^a^
KNIGHT	*Baseline*	*3 mo of sex hormone therapy*	*Baseline*	*3 mo of sex hormone therapy*
PUA, μmol/L	373 (±60)	314 (±57)^a^	271 (±53)	323 (±56)^a^
UAC, mL/min	6.3 (±1.8)	7.4 (±2.9)^c^	6.3 (±1.4)	5.3 (±1.5)^b^
FE-UA, %	5.0 (±1.4)	5.3 (±1.6)	5.9 (±1.1)	5.1 (±1.0)^a^

Unless otherwise indicated, median (interquartile range) is presented for non-normally distributed continuous variables and mean (±SD) is presented for normally distributed continuous variables. Significance is determined by linear mixed model comparing baseline and follow-up: a P < 0.001, b P < 0.01, c P < 0.05. Abbreviations: BMI, body mass index; PUA, plasma uric acid; UAC; uric acid clearance; FE-UA, fractional excretion of uric acid; ENIGI, European Network for the Investigation of Gender Incongruence; KNIGHT, Kidney fuNction In people receiving Gender-affirming Hormone Therapy.

### Uric acid metabolism

**Fig. 2:**
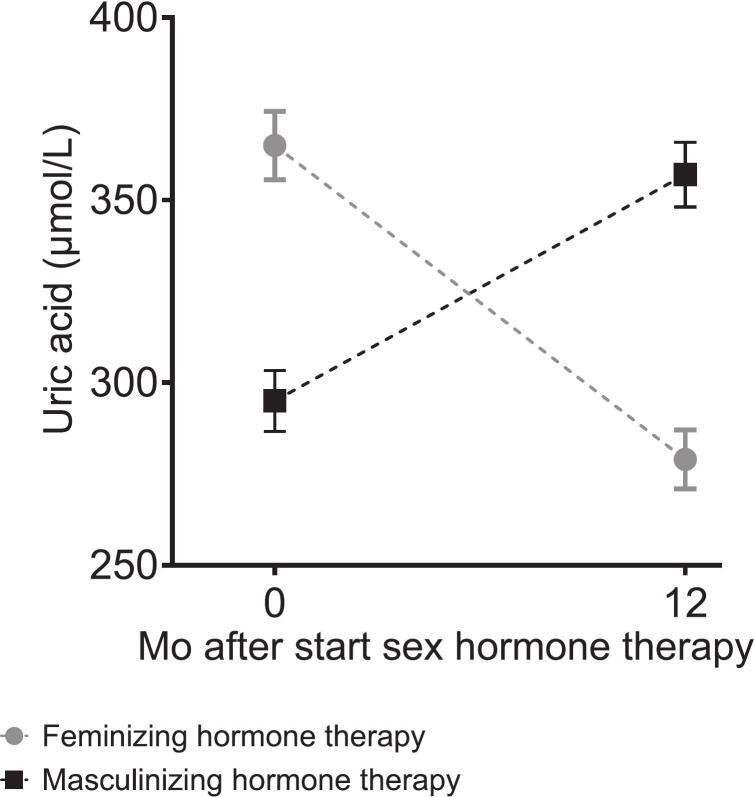
PUA changed during 1 year of sex hormone therapy in participants of the ENIGI study. Mean with 95% CI of PUA before and at 12 months of sex hormone therapy. Abbreviations: PUA, plasma uric acid; CI, confidence interval. Alt text: Graph shows mean (95% CI) plasma uric acid concentration at baseline and 12 months after starting feminizing or masculinizing hormone therapy.

**Fig. 3:**
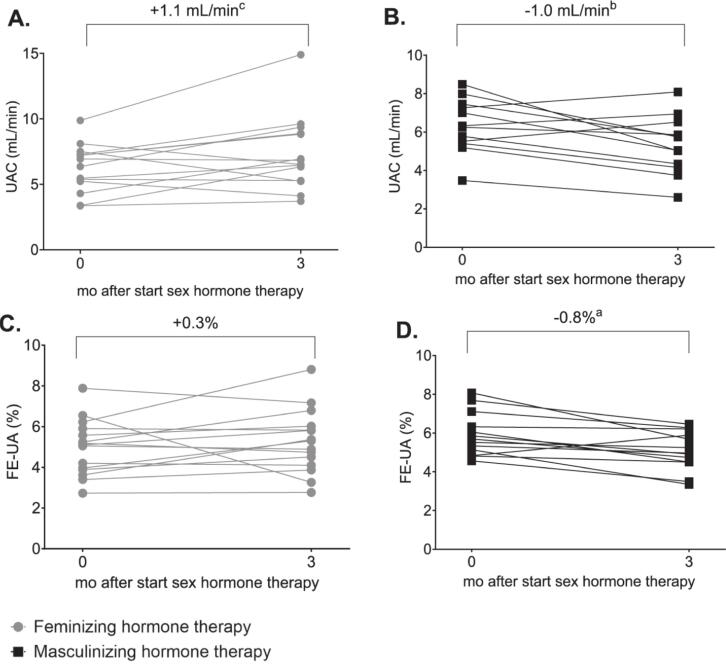
Change in uric acid clearance (A + B) and fractional uric acid excretion (C + D) in participants of the KNIGHT study during feminizing (A + C) and masculinizing (B + D) therapy. a, p < 0.001; b, p < 0.01; c, p < 0.05; Abbreviations: UAC, uric acid clearance; FE-UA, fractional excretion of uric acid. Alt text: Graphs A-D show individual UAC and FE-UA levels at baseline and 3 months after starting feminizing or masculinizing hormone therapy, with overall changes shown as percentages.

During feminizing hormone therapy, PUA decreased by 86 μmol/L (95% CI, 77 to 95) in ENIGI participants and 58 μmol/L (95% CI, 29 to 88) in KNIGHT participants. UAC increased (+1.1 mL/min; 95% CI, 0.1 to 2.1), while FE-UA remained unchanged (Table 2, Fig. 2, Fig. 3). Conversely, during masculinizing hormone therapy, PUA increased by 61 μmol/L (95% CI, 53 to 70) in ENIGI participants and 51 μmol/L (95% CI, 38 to 64) in KNIGHT participants, with reductions in both UAC (−1.0 mL/min; 95% CI, −1.7 to −0.3];) and FE-UA (−0.8%; 95% CI, −1.3 to −0.4; Table 2, Fig. 2, Fig. 3).

Delta PUA correlated inversely with delta serum estradiol and positively with delta serum testosterone, while delta FE-UA only correlated with delta serum testosterone (Table 3). Delta PUA also correlated with delta fat distribution ratios (android-to-gynoid, rho = 0.64; visceral-to-subcutaneous, rho = 0.32; both p < 0.001).

**Table 3:** Correlations between change in serum estradiol and testosterone and change in PUA, UAC and FE-UA.

Variables	Δ Serum estradiol	Δ Serum testosterone
	*R*	*R*
Δ PUA, μmol/L	−0.32^a^	0.63^a^
Δ UAC, mL/min	0.23	−0.38
Δ FE-UA, %	0.19	−0.53^b^

Correlation with PUA is calculated for the ENIGI cohort and correlation with UAC and FE-UA is calculated for the KNIGHT cohort. a, P < 0.001, b P < 0.01. Abbreviations: Δ, delta; R, Spearman’s rank correlation coefficient; PUA, plasma uric acid; UAC; uric acid clearance; FE-UA, fractional excretion of uric acid; ENIGI, European Network for the Investigation of Gender Incongruence; KNIGHT, Kidney fuNction In people receiving Gender-affirming Hormone Therapy.

Sensitivity analyses excluding individuals reporting current smoking or users of medications affecting uric acid metabolism did not alter these findings (Supplemental Table 3). Adjustments for delta BMI, blood pressure, and alcohol intake minimally influenced results; however, controlling for changes in lean body mass attenuated the magnitude of PUA change during masculinizing hormone therapy, while adjusting for total fat mass increased the magnitude of PUA change during feminizing hormone therapy (Supplemental Table 4). Adjusting for delta android-to-gynoid fat ratio and filtration markers (creatinine and cystatin C) attenuated but did not eliminate the observed changes (Supplemental Table 4). Estradiol administration route did not affect PUA. Testosterone undecanoate use was associated with a non-significant trend toward greater PUA increase compared to other testosterone formulations, particularly transdermal gel (Supplemental Table 5).

### Plasma proteomics

Feminizing and masculinizing hormone therapy were associated with 49 and 356 differentially expressed proteins (DEPs), respectively. These DEPs are discussed in more detail elsewhere [24]. Proteins involved in uric acid production (ADA, PNP, GUAD, XDH, HPRT, PRPS1, APT) showed no significant changes during sex hormone therapy (Supplemental Table 6).

#### Individual proteins associated with delta PUA

**Fig. 4:**
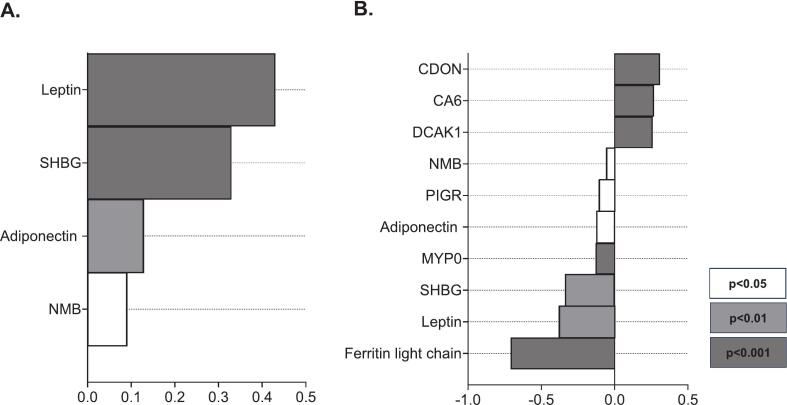
Log fold changes of the top 10 DEPs associated with changes in PUA during feminizing and masculinizing hormone therapy. The displayed proteins represent DEPs observed during feminizing (A) and masculinizing (B) hormone therapy, whose changes during sex hormone therapy (combining both feminizing and masculinizing therapies) were correlated with delta PUA. A Venn diagram summarizing the identification of these proteins can be found in Supplemental Fig. 3. P-values were adjusted to maintain a false discovery rate of 5%. Abbreviations: DEPs, differentially expressed proteins; PUA, plasma uric acid; SHBG, sex hormone-binding globulin; NMB, neuromedin-B; CDON, cell adhesion molecule-related/down-regulated by oncogenes; CA6, carbonic anhydrase 6; DCAK1, serine/threonine-protein kinase DCLK1; PIGR, polymeric immunoglobulin receptor; MYP0, myelin protein P0. Alt text: Graphs A and B log fold changes of the top 10 proteins associated with plasma uric acid changes during feminizing and masculinizing hormone therapy.

Across all participants, 350 proteins correlated with PUA changes; 126 of these proteins overlapped with the DEPs during feminizing or masculinizing hormone therapy (Supplemental Fig. 3; Supplementary Excel File 1) [24]. Among the top 10 DEPs that correlated with PUA changes, leptin, adiponectin, neuromedin-B (NMB), myelin protein P0 (MYP0), sex hormone-binding globulin (SHBG), polymeric immunoglobulin receptor (PIGR), and ferritin light chain negatively correlated with changes in PUA, while carbonic anhydrase 6 and DCLK1 showed positive associations (Table 4, Fig. 4).

**Table 4:** Summary of the top 10 DEPs associated with changes in PUA during sex hormone therapy.

Protein	Gene	Δ PUA
Δ Leptin	LEP	−0.77^a^
Δ Carbonic anhydrase 6	CA6	0.77^a^
Δ DCAK1	DCLK1	0.76^a^
Δ Adiponectin	ADIPOQ	−0.75^a^
Δ NMB	NMB	−0.75^a^
Δ MYP0	MPZ	−0.71^a^
Δ SHBG	SHBG	−0.71^a^
Δ PIGR	PIGR	−0.68^a^
Δ CDON	CDON	0.67^a^
Δ Ferritin light chain	FTL	−0.66^a^

Showing correlations between delta DEPs and delta PUA. P-values were adjusted to maintain a false discovery rate of 5%. a, P < 0.001. Abbreviations: Δ, delta; DEPs, differentially expressed proteins; DCAK1, serine/threonine-protein kinase DCLK1; NMB, neuromedin-B; MYP0, myelin protein P0; SHBG, sex hormone-binding globulin; PIGR, polymeric immunoglobulin receptor; CDON, cell adhesion molecule-related/down-regulated by oncogenes.

#### Ingenuity pathway analysis

Ingenuity Pathway Analysis (IPA) was conducted using the SOMAScan assay protein set as the reference. Feminizing hormone therapy led to 61 differentially expressed pathways, with the majority of the top 10 pathways being downregulated, especially those associated with protein synthesis and amino acid metabolism. In contrast, masculinizing hormone therapy resulted in 117 differentially expressed pathways, with most of the top 10 pathways upregulated, particularly those linked to extracellular matrix remodeling, tissue restructuring, and immune and inflammatory responses. These pathways are discussed in more detail elsewhere [24]. Here, we focused here on those correlated with delta PUA.

When combining individuals undergoing feminizing and masculinizing hormone therapy, changes in PUA was associated with the up- or downregulation of 75 pathways. When the analysis was limited to differentially expressed pathways specific to either feminizing or masculinizing hormone therapy, 30 pathways were found to correlate with changes in PUA (Supplemental Table 7; Supplementary Excel File 2). A Venn diagram summarizing the identification of these pathways is available in Supplemental Fig. 4. Notably, neural cell adhesion molecule (NCAM) signaling for neurite out-growth and integrin cell surface interactions were negatively correlated with PUA, while positively correlated pathways were mainly involved in protein synthesis and translation, signal transduction, cell stress and apoptosis, and cell structure.

## Discussion

This study investigated how uric acid metabolism changes during sex hormone therapy in two well-characterized transgender cohorts. Consistent with prior research, PUA decreased during feminizing hormone therapy and increased during masculinizing hormone therapy [17], [18], [19]. The increase in PUA during masculinizing hormone therapy appeared most pronounced among participants using intramuscular testosterone undecanoate. This difference was not explained by measured serum testosterone concentrations between administration routes (data not shown). A possible explanation is improved treatment adherence associated with long-acting injections, which may result in higher overall testosterone exposure over time.

The changes in PUA during sex hormone therapy were accompanied by altered kidney uric acid clearance and excretion: UAC increased during feminizing hormone therapy, whereas both UAC and FE-UA decreased during masculinizing hormone therapy, consistent with a prior study [17]. Additionally, changes in PUA were associated with changes in fat distribution during sex hormone therapy. Feminizing hormone therapy reduced, whereas masculinizing hormone therapy increased both the android-to-gynoid and visceral-to-subcutaneous fat ratios. Both these ratios were found to strongly correlate with changes in PUA. This aligns with earlier findings indicating that higher proportions of android and visceral fat, which are metabolically more active and pro-inflammatory, are associated with increased PUA concentrations [22], [32], [33], [34].

This hypothesis is further supported by our proteomic analysis, that revealed no changes in proteins directly involved in uric acid production, but identified strong associations between PUA changes and proteins linked to fat distribution, including adiponectin, leptin, and SHBG. All three proteins increased during feminizing hormone therapy and decreased during masculinizing hormone therapy and were inversely correlated with delta PUA. Given that lower adiponectin and SHBG concentrations are both associated with an increased android-to-gynoid ratio [35], [36], [37], [38], [39], [40], [41], [42], [43], [44], and increased visceral fat [45], [46], [47], and higher leptin concentrations are associated with increased subcutaneous fat [48], [49], [50], these proteomic findings add to the hypothesis that the redistribution of fat during sex hormone therapy may play an indirect role in influencing PUA concentrations.

Regarding adiponectin, the negative association with uric acid aligns with previous studies [51], [52], [53], [54]. Further emphasizing the association between adiponectin, visceral fat and PUA, is a study in severely obese patients that demonstrated that adiponectin gene expression in VAT negatively correlated with serum concentrations of uric acid [55]. In addition to indirect effects via fat distribution, adiponectin may also influence uric acid production directly, as adiponectin was shown in a previous study to be inversely associated with xanthine oxidase activity [56]. Regarding leptin, the inverse correlation with PUA contrasts with most previous cross-sectional studies [57], [58], [59], [60], [61], [62], [63], [64]. Regarding SHBG, the association with PUA is consistent with previous studies [65], [66], [67], [68]. This association may be due to changes in fat distribution, affecting both SHBG and PUA. However, an alternative explanation is that elevated PUA itself affects SHBG through inactivation of AMP-activated protein kinase (AMPK), which has been shown to impair liver metabolism and reduce SHBG production [66]. Together, these findings suggest that fat redistribution due to sex hormones may play a role in sex hormone-induced changes in PUA concentrations.

The clinical importance of these findings lies in the well-established association between higher PUA and CKM-related conditions [1], [8], [69], [70], [71]. Sex hormone-driven differences in PUA may partly explain sex disparities in these conditions, especially the relative protection seen in premenopausal women [8], [9], [10], [11], [12], [13]. Given uric acid’s association with CKM, it is an important shared biomarker and therapeutic target that has been explored in a number of clinical trials [72], [73], [74], [75], [76], [77], [78], [79]. Although prior studies targeting uric acid production with allopurinol failed to significantly influence CKD progression, these trials were limited by low hyperuricemia prevalence and insufficient power. Furthermore, emerging evidence suggests that promoting uric acid excretion may be critical for therapeutic efficacy [80].

While the use of two complementary cohorts, the large ENIGI cohort and the mechanistic KNIGHT cohort, strengthens the validity and interpretation of our findings, several limitations warrant consideration. The follow-up durations were limited to 3 months in KNIGHT and 12 months in ENIGI, restricting insights into the long-term effects of sex hormone therapy on uric acid metabolism. Additionally, individuals with pre-existing CKD or cardiovascular disease were excluded, preventing generalization to these populations. Moreover, both cohorts were predominantly recruited from a single geographic region, which may limit external validity. Furthermore, the feminizing regimens combined estradiol with antiandrogens (CPA or triptorelin), complicating isolation of estradiol-specific effects. In addition, standardizing protein and salt intake, and correcting for alcohol intake may not fully account for dietary influences on PUA, as other factors such as purine source, and fructose intake also play an important role. Also, longitudinal DXA-derived changes in body composition and fat distribution may have been influenced to some extent by software updates. Finally, key urate transporters involved in kidney and intestinal excretion could not be assessed via plasma proteomics.

In conclusion, this study demonstrates that PUA concentrations decrease during feminizing hormone therapy and increase during masculinizing hormone therapy. These changes were associated with alterations in uric acid clearance/excretion and with sex hormone–related changes in body fat distribution. These insights help elucidate the role of sex hormones in the shared pathophysiology of cardiovascular, kidney, and metabolic conditions, informing more personalized therapeutic strategies to lower PUA in CKM-related diseases and ultimately enhancing care for both transgender and cisgender individuals.

## CRediT authorship contribution statement

**Sarah A. van Eeghen:** Methodology, Project administration. **Danii L.S. Suijk:** Writing – review & editing. **Laura L. Pyle:** Writing – review & editing, Visualization, Formal analysis. **Phoom Narongkiatikhun:** Writing – review & editing. **Ye Ji Choi:** Writing – review & editing. **Natalie J. Nokoff:** Writing – review & editing, Resources, Project administration, Investigation. **Petter Bjornstad:** Writing – review & editing, Resources. **Martin den Heijer:** Writing – review & editing, Conceptualization. **Daniël H. van Raalte:** Supervision, Conceptualization, Resources, Methodology, Project administration, Writing – review & editing.

## Funding

This research did not receive any specific grant from any funding agency in the public, commercial or not-for-profit sector.

## Declaration of competing interest

The authors declare the following financial interests/personal relationships which may be considered as potential competing interests: PB reports serving or having served as a consultant for AstraZeneca, Bayer, Bristol-Myers Squibb, Boehringer Ingelheim, Eli-Lilly, LG Chemistry, Sanofi, Novo Nordisk, and Horizon Pharma. PB also serves or has served on the advisory boards and/or steering committees of AstraZeneca, Bayer, Boehringer Ingelheim, Novo Nordisk, and XORTX. PB has received operational funding for clinical trials from Boehringer Ingelheim, Lilly, Merck, AstraZeneca, Horizon Pharma/Amgen and Novo Nordisk. NJN is a consultant for Neurocrine Biosciences and an expert panel member for World Athletics. DHR is an Deputy Editor for the European Journal of Endocrinology.
