# Supplementary material for: Changes in uric acid metabolism and associated plasma proteomics during sex hormone therapy
Source: J Clin Transl Endocrinol. 2026 Feb 27;44:100434. doi: 10.1016/j.jcte.2026.100434 (PMC12995700; doi:10.1016/j.jcte.2026.100434)
Supplement: Supplementary Data 3 [file mmc3.docx]

**Supplemental Material**

Table of contents: Page

Supplemental Table 1 2

Supplemental Table 2 3

Supplemental Table 3 4

Supplemental Table 4 5

Supplemental Table 5 6

Supplemental Table 6 7

Supplemental Table 7 8

Supplemental Figure 1 10

Supplemental Figure 2 11

Supplemental Figure 3 12

Supplemental Figure 4 13

Supplemental Excel files 14

| Feminizing hormone therapy | ENIGI (N= 260) | KNIGHT (N=15) |
| --- | --- | --- |
| Estradiol |  |  |
| Transdermal estradiol, n (%) | 136 (52) | 14 (93%) |
| Oral estradiol, n (%) | 118 (45) | 1 (7) |
| Only CPA during first 3 months, n (%) | 6 (2) | 0 (0) |
| Antiandrogen |  |  |
| Oral CPA, n (%) | 257 (99) | 0 (0) |
| IM triptorelin, n (%) | 0 | 15 (100) |
| No antiandrogen, n (%) | 3 (1) | 0 (0) |
| Masculinizing hormone therapy | **ENIGI (N= 284)** | **KNIGHT (N=13)** |
| Transdermal testosterone gel, n (%) | 140 (49%) | 12 (92) |
| IM testosterone undecanoate, n (%) | 33 (12) | 0 (0) |
| IM testosterone blend, n (%) | 111 (39) | 1 (8) |

**Supplemental Table 1. Participants started with different administration routes for feminizing and masculinizing hormone therapy**

Abbreviations: ENIGI, European Network for the Investigation of Gender Incongruence; KNIGHT, Kidney fuNction In people receiving Gender Affirming Hormone Therapy; CPA, cyproterone acetate; IM, intramuscular

| Serum sex hormones | During feminizing hormone therapy | | During masculinizing hormone therapy | |
| --- | --- | --- | --- | --- |
| ENIGI | *Baseline* | *12 mo of HT* | *Baseline* | *12 mo of HT* |
| Testosterone, nmol/L | 18 (14-24) | 0.6 (0.5-0.9)^a^ | 1.3 (0.9-1.6) | 25 (17-37)^a^ |
| Estradiol, pmol/L | 99 (75-118) | 224 (151-347)^a^ | 175 (76-370) | 161 (115-218) |
| KNIGHT | *Baseline* | *3 mo of HT* | *Baseline* | *3 mo of HT* |
| Testosterone, nmol/L | 15.0 (8.4-20) | 0.5 (0.4-0.6)^a^ | 1.0 (0.8-1.1) | 23 (11-41)^a^ |
| Estradiol, pmol/L | 74 (60-94) | 242 (180-301)^a^ | 137 (93-302) | 158 (120-239) |

**Supplemental Table 2. Serum sex hormone concentrations before and during sex hormone therapy**

Data are presented according to their distribution; median (interquartile range).Significant changes between baseline and 12 months of sex hormone therapy: a P<0.001, b P<0.01, c P<0.05.

Abbreviations: ENIGI, European Network for the Investigation of Gender Incongruence; KNIGHT, Kidney fuNction In people receiving Gender Affirming Hormone Therapy; HT, hormone therapy

**Supplemental Table 3. Change in PUA (μmol/L; 95% CI) after 12 months of sex hormone therapy in participants of the ENIGI study, after several sensitivity analyses**

|  | During feminizing hormone therapy | During masculinizing hormone therapy | |
| --- | --- | --- | --- |
| PUA, μmol/L | -86 (-95 to -77) | | +61 (53 to 70) |
| Excluding subjects reporting current smoking: | -89 (-100 to -78) | | +63 (53 to 74) |
| Excluding subjects using: |  | |  |
| Finasteride/Dutasteride | -86 (-95 to -76) | |  |
| Progestogens/other contraceptives |  | | +65 (55 to 74) |
| thiazide diuretics | -86 (-95 to -77) | | +61 (53 to 70) |
| Aspirin | -86 (-95 to 78) | | +61 (52 to 70) |
| Antibiotics | -86 (-95 to -77) | | +61 (53 to 70) |
| Allopurinol | -86 (-95 to -77) | | +61 (53 to 70) |
| Nifedipine | -86 (-95 to -77) | | +61 (53 to 70) |
| Propranolol | -86 (-95 to -77) | | +61 (52 to 69) |
| All above | -86 (-95 to -77) | | +64 (54 to 74) |

Abbreviations: PUA, plasma uric acid; ENIGI, European Network for the Investigation of Gender Incongruence

**Supplemental Table 4. Change in PUA (μmol/L; 95% CI) after 12 months of sex hormone therapy in participants of the ENIGI study, after correction for change (Δ) in body mass index, blood pressure, serum creatinine, serum cystatin C, and alcohol consumption, and % change in lean body mass and fat mass.**

|  | During feminizing hormone therapy | During masculinizing hormone therapy | |
| --- | --- | --- | --- |
| PUA, μmol/L | -86 (-95 to -77) | | +61 (53 to 70) |
| Corrected for |  | |  |
| Δ BMI, kg/m^2^ | -92 (-101 to -83) | | +62 (53 to 71) |
| Δ systolic BP, mmHg | -88 (-98 to -78) | | +63 (53 to 72) |
| Δ diastolic BP, mmHg | -89 (-99 to -79) | | +63 (53 to 72) |
| Δ creatinine, μmol/L | -74 (-86 to -62) | | +39 (24 to 54) |
| Δ cystatin C, mg/L | -68 (-75 to -60) | | +47 (40 to 64) |
| % change in lean body mass | -82 (-97 to -66) | | +48 (20 to 76) |
| % change in total fat mass | -109 (-128 to -90) | | +67 (50 to 85) |
| Δ android-to-gynoid fat ratio | -77 (-98 to -56) | | +53 (32 to 74) |
| Δ alcohol consumption | -89 (-99 to -80) | | +63 (54 to 72) |

Abbreviations: ENIGI, European Network for the Investigation of Gender Incongruence; PUA, plasma uric acid; BMI, body mass index; BP, blood pressure; Δ, delta;

| Variable | During feminizing hormone therapy | n | During masculinizing hormone therapy | n |
| --- | --- | --- | --- | --- |
| PUA, μmol/L, exclusion of participants changing administration route during the first year of sex hormone therapy | -85 (-94 to -75) | 233 | +57 (47 to 67) | 208 |
| PUA, μmol/L, for different administration routes |  |  |  |  |
| IM testosterone (blend injection or undecanoate injection) |  |  | +60 (46 to 74) | 119 |
| *Blend injection* |  |  | +54 (39 to 69) | 93 |
| *Undecanoate injection* |  |  | +80 (51 to 110) | 26 |
| Transdermal testosterone (gel) |  |  | +53 (38 to 68) | 89 |
| Oral estradiol valerate | -89 (-103 to -74) | 114 |  |  |
| Transdermal estradiol (gel and patch) | -81 (-94 to -68) | 119 |  |  |

**Supplemental Table 5. Change in PUA (μmol/L; 95% CI) after 12 months of sex hormone therapy in participants of the ENIGI study, for different administration routes**

Abbreviations: PUA, plasma uric acid; IM, intramuscular

**Supplemental Table 6. Log fold changes (P-value) of proteins involved in uric acid metabolism during feminizing and masculinizing hormone therapy.**

| Protein | During feminizing hormone therapy | During masculinizing hormone therapy |
| --- | --- | --- |
| ADA | -0.027 (p=0.63) | -0.032 (p=0.45) |
| PNP | -0.115 (p=0.58) | 0.238 (p=0.36) |
| GUAD | -0.073 (p=0.55) | -0.037 (p=0.39) |
| XDH | 0.013 (p=0.47) | -0.045 (p=0.14) |
| HPRT | -0.063 (p=0.60) | 0.072 (p=0.71) |
| PRPS1 | -0.022 (p=0.80) | -0.005 (p=0.91) |
| APT | -0.089 (p=0.70) | 0.122 (p=0.74) |

P-values were adjusted to maintain a false discovery rate of 5%.

Abbreviations: ADA, adenosine deaminase; PNP, purine nucleoside phosphorylase; GUAD, guanine deaminase; XDH, xanthine dehydrogenase/oxidase; HPRT, hypoxanthine-guanine phosphoribosyltransferase; PRPS1, ribose-phosphate pyrophosphokinase 1; APT, adenine phosphoribosyltransferase

| Ingenuity Canonical Pathways | Z-scores (-log[p-value]) | |
| --- | --- | --- |
|  | *During feminizing hormone therapy* | *Δ PUA correlation* |
| Eukaryotic Translation Initiation | -4.00 (4.20) | 4.49 (3.23) |
| EIF2 Signaling | -1.60 (3.55) | 2.83 (2.47) |
| Pulmonary Healing Signaling Pathway | 1.62 (1.81) | 0.42 (2.35) |
| Regulation of eIF4 and p70S6K Signaling | -1.41 (1.31) | 0.63 (2.30) |
| Eukaryotic Translation Elongation | -3.74 (5.65) | 4.03 (2.17) |
| Eukaryotic Translation Termination | -3.61 (5.07) | 3.90 (2.00) |
| Nonsense-Mediated Decay (NMD) | -3.61 (3.70) | 3.55 (1.94) |
| MSP-RON Signaling in Cancer Cells Pathway | -1.23 (2.08) | 1.92 (1.82) |
| ERCC6 (CSB) and EHMT2 (G9a) positively regulate rRNA expression | -1.0 (1.84) | 1.89 (1.69) |
| Response of EIF2AK4 (GCN2) to amino acid deficiency | -3.74 (4.39) | 2.99 (1.51) |
| Hepatic Fibrosis / Hepatic Stellate Cell Activation | # (1.74) | # (1.45) |
| Macropinocytosis Signaling | -1.51 (1.87) | 1.34 (1.42) |
| Regulation of the Epithelial-Mesenchymal Transition Pathway | # (1.77) | # (1.40) |
| mTOR Signaling | -1.90 (2.08) | 2.47 (1.37) |
|  | *During masculinizing hormone therapy* | *Δ PUA correlation* |
| Intrinsic Pathway for Apoptosis | 2.50 (1.43) | 3.02 (2.37) |
| Pulmonary Healing Signaling Pathway | -1.24 (1.92) | 0.42 (2.35) |
| Regulation of eIF4 and p70S6K Signaling | 0.54 (1.55) | 0.63 (2.30) |
| G Beta Gamma Signaling | 1.63 (2.10) | 3.89 (2.08) |
| Translocation of SLC2A4 (GLUT4) to the plasma membrane | 2.31 (1.65) | 3.44 (2.00) |
| Integrin signaling | 2.53 (2.31) | 2.57 (1.97) |
| Insulin Receptor Signaling | 0.56 (1.44) | 1.73 (1.82) |
| MSP-RON Signaling in Cancer Cells Pathway | -0.82 (1.41) | 1.92 (1.82) |
| Oxidative Stress Induced Senescence | 0.69 (2.69) | 1.00 (1.79) |
| Costimulation by the CD28 family | 0.94 (1.60) | 1.30 (1.67) |
| UVC-Induced MAPK Signaling | 0.50 (1.64) | 1.96 (1.66) |
| RET signalin | 1.16 (1.49) | 1.15 (1.65) |
| Signaling by ERBB2 | 0 (1.76) | 1.98 (1.47) |
| NUR77 Signaling in T Lymphocytes | -0.24 (1.45) | 0.56 (1.47) |
| CLEAR Signaling Pathway | 0.38 (2.00) | 0.60 (1.46) |
| Colorectal Cancer Metastasis Signaling | -0.15 (1.70) | 0.56 (1.45) |
| Hepatic Fibrosis / Hepatic Stellate Cell Activation | # (5.1) | # (1.45) |
| NCAM signaling for neurite out-growth | -0.45 (3.32) | -0.20 (1.41) |
| IL-4 Signaling | 0.31 (3.43) | 1.17 (1.40) |
| Regulation of the Epithelial-Mesenchymal Transition Pathway | # (1.30) | # (1.40) |
| Integrin cell surface interactions | -0.96 (2.62) | -0.97 (1.30) |

**Supplemental Table 7. Z-scores of differentially expressed pathways during feminizing and masculinizing hormone therapy, and the correlation of these pathway with Δ PUA.**

Pathways that are listed are the ones that were significantly upregulated or downregulated during feminizing or masculinizing hormone therapy, where the changes during sex hormone therapy (considering feminizing and masculinizing therapies combined) were associated with Δ PUA; # no activity pattern available. Abbrevations: Δ, delta; PUA, plasma uric acid; EIF2, eukaryotic initiation factor 2; eIF4, eukaryotic initiation factor 4; p70S6K, 70-kDa ribosomal protein S6 kinase; NMD, nonsense-mediated mRNA decay; MSP, macrophage-stimulating protein; RON, Recepteur d’Origine Nantais (a receptor tyrosine kinase); ERCC6 (CSB), excision repair cross-complementation group 6 (Cockayne syndrome group B); EHMT2 (G9a), euchromatic histone lysine methyltransferase 2; EIF2AK4 (GCN2), eukaryotic translation initiation factor 2 alpha kinase 4 (general control nonderepressible 2); mTOR, mechanistic target of rapamycin; SLC2A4 (GLUT4), solute carrier family 2 member 4 (glucose transporter type 4); MAPK, mitogen-activated protein kinase; ERBB2, Erb-B2 receptor tyrosine kinase.

**
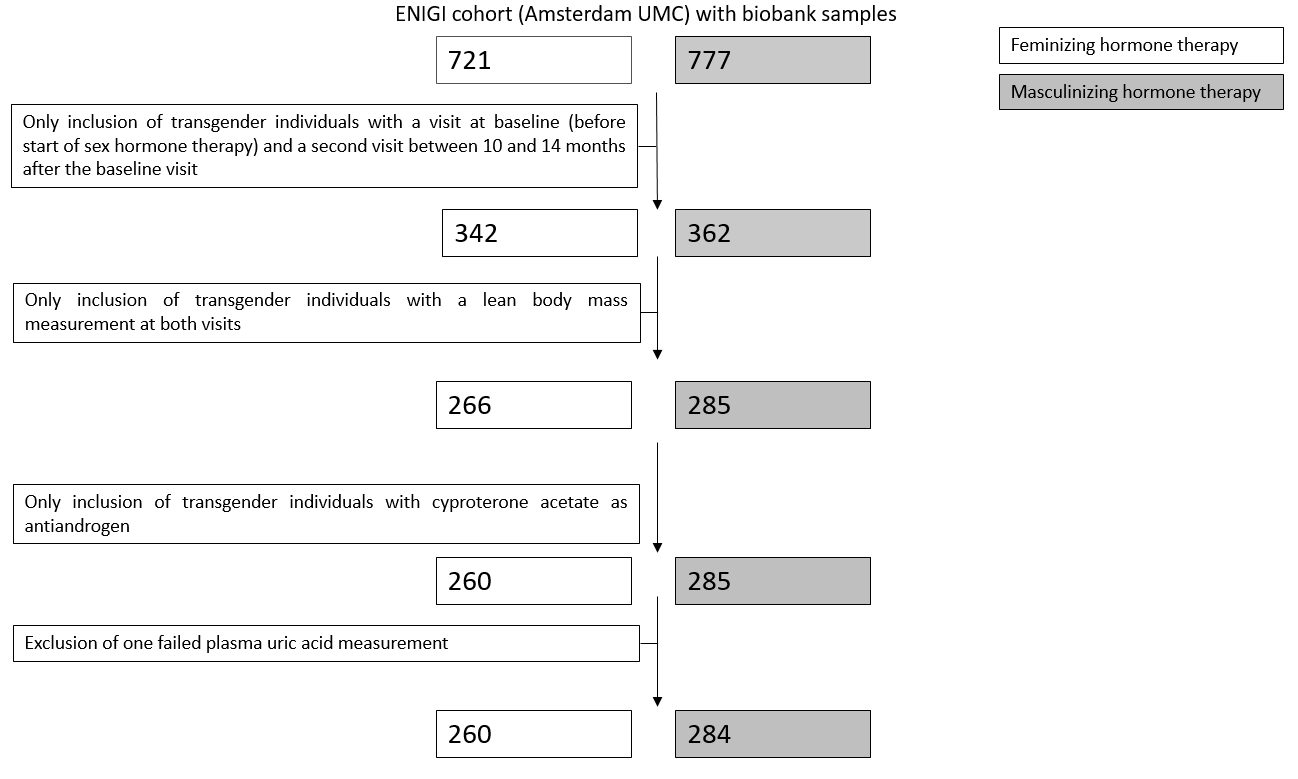
Supplemental figure 1.** Flowchart of the inclusion process ENIGI

Abbreviations: ENIGI, European Network for the Investigation of Gender Incongruence; UMC, university medical center


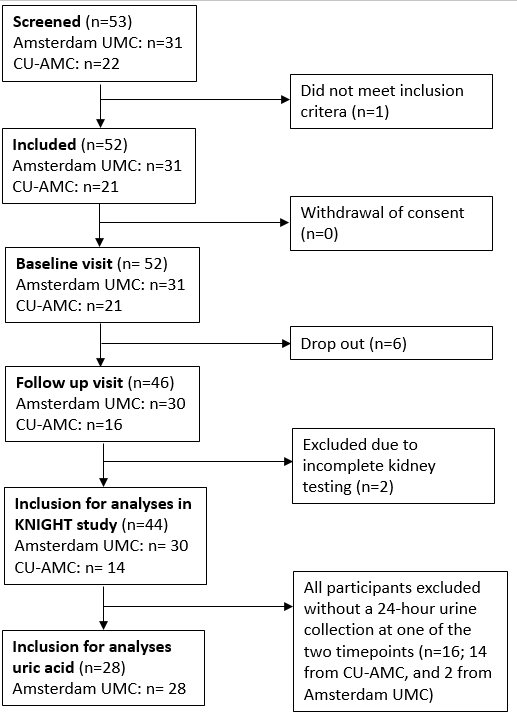


**Supplemental figure 2.** Flowchart of the inclusion process KNIGHT

Abbreviations: KNIGHT, Kidney fuNction In people receiving Gender Affirming Hormone Therapy; UMC, university medical center; CU-AMC, University of Colorado Anschutz Medical Campus


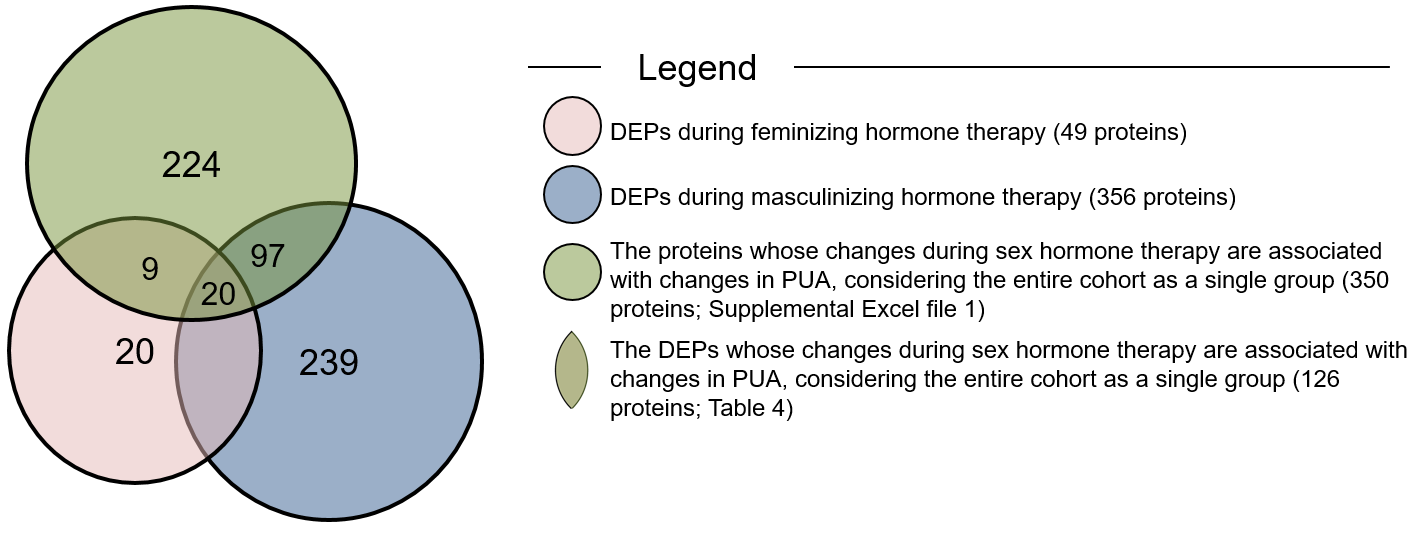


**Supplemental Figure 3.** Identification of DEPs whose changes during sex hormone therapy are associated with changes in PUA during sex hormone therapy considering the entire cohort as a single group.

Abbreviations: DEPs, differentially expressed proteins; PUA, plasma uric acid


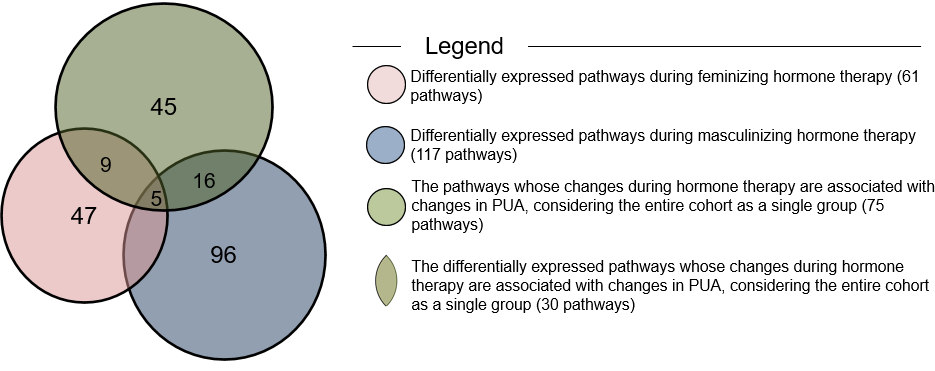


**Supplemental Figure 4.** Identification of differentially expressed pathways whose changes during hormone therapy are associated with changes in PUA during hormone therapy, considering the entire cohort as a single group.

Abbreviations: PUA, plasma uric acid

**Supplemental excel files:**

Supplemental Excel file 1: Correlations of changes in proteins with changes in plasma uric acid

Supplemental Excel file 2: Correlations of changes in pathways with changes in plasma uric acid
